# The Hirsch index - a play on numbers or a true appraisal of academic output?

**DOI:** 10.1186/1755-7682-4-25

**Published:** 2011-07-07

**Authors:** Taimur Saleem

**Affiliations:** 1 Medical College, Aga Khan University, Stadium Road, Karachi 74800, Pakistan

## Abstract

Citation metrics have rapidly gained importance in today's landscape and are being increasingly utilized as a yardstick in making several important decisions regarding academic funding and appointments. The impact factor has traditionally been the metric most often employed in this regard. However, the emergence of the Hirsch index has provided an alternative to the impact factor. The h-index, despite its flaws, continues to gain acceptance and popularity in the medical community. Several medical journals have evaluated and endorsed the use of the h-index. However, it must be interpreted with all of its limitations in mind.

## Introduction

Over the years, different citation indices and bibliometric parameters have been formulated to measure academic output and scholarly activity of researchers and academicians across the globe. The importance of these metrics springs from the fact that various agencies bequeath grants and funds on the basis of these indices. Thus they influence the amount, structure and orientation of research allocation and endorsement [1]. In a dynamic world with limited resources [2], the importance of such bibliometric measures cannot, therefore, be denied. These indices are also used as a foundation for conferring academic awards, hierarchal promotions, tenures, fellowships, salary increments, recruitments and leadership positions [2-5]. These indices are surrogate markers of scientific output [6] and ultimately demonstrate how "efficient" and "effective" a journal is [7]. They can also be considered a self-regulated effort to enforce some measure of quality control in a rapidly growing industry [6].

Citation metrics heavily influence the trends of subscription for various scientific journals and serve as a yardstick for authors when they deliberate about where to submit their next best scientific works [7]. According to Ogden et al, "the attraction of having a simple single number to judge complex issues is too great"[7]. Despite their potential shortcomings, some of these citation metrics continue to garner immense momentum and acceptance in the scientific world.

## Impact factor

Traditionally, the citation metric most often employed in medical circles to evaluate a journal's standing is the impact factor (IF). The IF, first proposed by Eugene Garfield and Irving Sher in 1963 [3], is published by Thomson Scientific Reuters on an annual basis in the Journal Citation Reports (JCR)[1]. The rationale behind the original derivation of IFs was based on their utility in the ease of selection of journals in the Science Citation Index (SCI)[7]. The IF of a journal for a particular year is calculated as "a ratio of the number of research papers cited from that journal to the total number of "citable" papers it has published, using the time bracket of previous two years"[4]. As such, several factors influence the IF including, but not limited to, the type of article, the discipline of research and the number of "citable" articles a journal publishes [3].

Table 1 lists the potential problems with IF usage in the contemporary medical landscape [3,4,7-9]. The IF has been criticized because of its propensity for "biased selection" [10,11]. Although having an over-simplified measure such as the IF leases convenience to bioscientists, one cannot deny the fact that this situation is actually akin to a stock exchange where major shareholders, or highly cited medical journals, wield a disproportionate degree of influence or monopoly on the rest of the investors in the industry [10].

**Table 1:** Pitfalls of using impact factor [3,4,7-9]

Pitfall	Detail
Transparency issues	Thompson Scientific decides what is a "citable" research item. Books, book chapters and conference proceedings not included.

Ambiguity in calculation	"The definition for citing items is broader than for cited items". Also, difficult to reproduce the calculation.

Shortcomings of 2 - year temporal bracket for citations	Citation fluctuations make the inclusion of citations limited to the previous two years unreliable.

No consideration of "citation half-life" in calculating IF ǂ	Citations change as the article and the journal itself matures.

Over-representation	Medical literature in English language or by a particular publisher or from a particular geographic region (e.g. North America) or about a particular subject (e.g. basic sciences) or of a particular type (e.g.a review) is disproportionately represented and cited.

Homonymy, Synonymy	Many authors sharing the same name or one article with many variants. Articles where author's name misspelled will be missed.

Delays	Delays in registration of citations, delays in peer-review and publication process detrimental for journals of disciplines with long turn-over times.

Abridged referencing	Many journals restrict the number of articles that can be cited by authors. Important research maybe potentially disregarded and not cited.

Miscellaneous	"Gift" authorships, self-citations and "flattery" citations

ǂ IF = Impact Factor

## The h-index

In order to circumvent these pitfalls associated with the usage of IF, the h-index was proposed as a bibliometric tool for individual authors, research groups or journals [7]. Most frequently, the h-index is utilized as an indicator of "individual scientific achievement" [9] because a "book should not be judged by its cover"[12].

Physicist Jorge E. Hirsch at the University of California, San Diego, first explained the calculation of the h-index in 2005 in his seminal publication as follows: "a scientist has the index h if h of his or her N_p _papers have at least h citations each and the remainder of the papers (N_p _- h) have ≤ h citations each, where N_p _is the number of papers published over n years"[2]. For example, if a scientist has an h-index of 70, it stands to follow that he has published atleast 70 papers with at least 70 citations each. Hirsch also listed some caveats that need to be considered when using the h-index (Table 2)[2,9]. Using the h-index as a guidepost, Hirsch also proposed the following criteria for faculty promotions at major research institutions, a) advancement to associate professor/tenure at h ≈ 12, b) advancement to full professor at h ≈ 18, c) fellowship or membership of major academic or professional societies may be granted at h ≥ 15 [2]. Scholars can calculate their h-index at search engines such as the ISI Web of Science [13], Elsevier's Scopus [14] or Google Scholar [15].

**Table 2:** Caveats with the use of h-index outlined by Hirsch [2,9]

#	Caveats
1.	A single number such as the h-index only tells a part of the story and never the whole story.

2.	Researchers in non-stream fields will not achieve very high h-indices.

3.	Skewness in the distribution of citations possible; affects the representativeness of the h-index.

4.	A scientist with a few but very highly cited papers will still have a low h-index.

5.	Increased collaborations likely to inflate the h-value.

6.	Self-citations can increase the h-index. This effect is more pronounced at lower h-indices.

7.	Senior authors and seasoned researchers likely to have a higher h-index when compared to their junior colleagues.

In a second paper by Hirsch in 2007, the h-index was shown to be a better predictor of future scientific performance and productivity when compared to other measures such as total number of citations, mean citations per paper and total number of papers [9]. Additionally, Hirsch argued that the h-index is fashioned in such a way that it predicts individual cumulative achievement better than other indices in a differential manner, as is the case for a paper with multiple co-authors with varying levels of seniority. On the other hand, indices such as Egghe's *g *index, Jin et al.'s AR index and Komulski's H^2 ^index are inferior because they indiscriminately credit a highly cited paper to all its co-authors [9]. In contrast to the championing of the h-index by Hirsch, Lehmann et al [16] reported the superiority of the mean number of citations per paper over the h-index [9]. Similarly, Honekopp et al [12] showed that the IF outperforms the h-index when predicting the future citations of an article.

Another issue with the h-index that merits discussion is the inability of the metric to differentiate between scientists who publish but their work goes "uncited" and researchers who publish little to begin with [6]. The same can be considered as an advantage of the h-index in that the authors with "practically" similar research profiles have similar h-indices [6].

Several journals have now evaluated the value of the h-index as a viable citation metric including Retrovirology [17,18], Journal of Neurosurgery [19-21], European Archives of Oto-Rhino-Laryngology [22], Academic Radiology [23], Breast Cancer Research and Treatment [24], Harvard Review of Psychiatry [25] and Urology [26]. These studies have shown that the h-index is a robust metric to evaluate scientific research in these disciplines.

The h-index has been described as being inherently biased towards more seasoned researchers with long spanning career trajectories [27,28]. It appears to favor researchers who publish in fields with high citation frequencies [1]. Gender also appears to have an impact on the h-index via its influence on productivity [27,28]. For developing countries, the additional problem posed in the computation of the h-index is that many authors heavily publish in local and national journals which may not be tracked by or be indexed in common scientific search engines or databases [29]. This may distort the calculation of the h-index for these researchers as these local journals may have very limited "citability" and circulation [29]. There is thus a need to make the journals in developing countries more stream-lined [5]. Till that time, it seems unfair to compare the scientific achievements of a scientist based in a developing country with those of a scientist in the developed world solely by the use of the h-index [5].

Although the h-index is considered to perform at a superior level when considering scientists in the same scientific category, there are exceptions to the rule particularly when one considers the possibility of the existence of subcategories within a category [5]. Like the IF, this index also does not consider books, book chapters or conference proceedings in its computation [5]. The h-index is also not sensitive for changes in academic performance and it can never decrease [30].

An additional measure, the e-index, has also been introduced more recently to provide more information about a researcher's published works [31,32]. Dodson believed that the h-index underestimates the actual number of citations by as much as 50%[31,33]. The e-index refers to the "further impact" that scientists have over and above the h-index. This is the "excess" citations that a researcher has in addition to those that have been used to compute the h-index [31,32]. To counter the effect of the author's age on the h-index, an m-score has been proposed which is calculated as "the h-index divided by the number of years since the first publication"[6,28]. In addition, a "mentoring index" has also been suggested [18] to give due and weighted credit to both senior and junior scientists who are the mentors and mentees respectively.

## Future directions

There is a need to find a balance between the quantitative and qualitative aspects of bibliometric indices. Additionally, there is a need to recognize the importance of expert opinion and review panels in deciding the scientific value of an article in addition to the numeric value assigned through a bibliometric index [34]. Despite its myriad advantages, a single index cannot, for all realistic purposes, be the ultimate authority on the scientific integrity and quality of scientific research [8]. More and more importance is being attached to bibliometric indices now and we need a modus operandi that is fair, honest, sophisticated, up-to-date, systematic and effective [1,10].

The h-index is certainly a step in the right direction. However, the verdict on the superiority of the h-index over other metrics is not unanimous at the moment, although positive trends can be well appreciated. This disputation invites further studies which may be best directed at the evaluation of the original h-index instead of derivation of further variants that will only add to the complexity of the problem [35]. Such studies should also provide a measure of concentration like the Gini coefficient or the Herfindahl index [35].

The importance of evaluating and rectifying flaws in current citation practices also needs to be highlighted. In this vein, journals can adopt the practice of random citation auditing [36]. Also, current bibliometric indices such as the h-index attach weight to frequency of citation. However, some authors have proposed that high readership be taken into account instead to gauge the true impact of a research paper [18].

## Conclusions

When compared to similar bibliometric indices, the h-index undoubtedly represents an appealing prospect. It has quickly emerged to become a powerful nucleus in the universe of citation metrics that has captured the attention and fascination of researchers and scientists worldwide [37]. It is also being considered as a sustainable alternative to the IF. Although not the quintessential or gold-standard citation metric, the h-index still represents one of the most holistic and realistic indicators of a researcher's credentials. It attempts to accord importance to both the quantity (number of publications) and quality (number of citations) of scientific research [38].

However, at the same time, we need to be mindful that like any other citation metric that maybe excessively relied upon in this climate of financial uncertainty [31] and increasing competition [5], it can engender a vicious cycle of "publish or perish", "adapt or perish", "citation game-playing" or "citation coalitions and networks" [5,36,39,40] while also quelling creative bravado [41]. It must, therefore, be interpreted cautiously in the backdrop of all of its limitations [27,37]. At the end of the day, the h-index essentially remains the corollary of complex statistical jargon. It remains a matter of intense debate whether scientific talent can truly be quantified by a numerical entity such as the h-index [41].

## Competing interests

The author declares that they have no competing interests.

## Authors' contributions

TS was involved in the conception, writing and final approval of the manuscript.

## References

[B1] Moed HF New developments in the use of citation analysis in research evaluation Arch Immunol Ther Exp (Warsz) 2009 57 13 8 10.1007/s00005-009-0001-5 19219533

[B2] Hirsch JE An index to quantify an individual's scientific research output Proc Natl Acad Sci USA 2005 102 16569 72 10.1073/pnas.0507655102 16275915 PMC1283832

[B3] Grzybowski A The journal impact factor: how to interpret its true value and importance Med Sci Monit 2009 15 SR1 4 19179983

[B4] Saleem T Impact factor - a pandora's box J Pak Med Assoc 2009 59 499 500 19579748

[B5] Kellner AW Ponciano LC H-index in the Brazilian Academy of Sciences: comments and concerns An Acad Bras Cienc 2008 80 771 81 10.1590/S0001-37652008000400016 19039498

[B6] Sebire NJ H-index and impact factors: assessing the clinical impact of researchers and specialist journals Ultrasound Obstet Gynecol 2008 32 843 5 10.1002/uog.6266 19035541

[B7] Ogden TL Bartley DL The ups and downs of journal impact factors Ann Occup Hyg 2008 52 73 82 10.1093/annhyg/men002 18316351

[B8] Ha TC Tan SB Soo KC The journal impact factor: too much of an impact? Ann Acad Med Singapore 2006 35 911 6 17219007

[B9] Hirsch JE Does the H index have predictive power? Proc Natl Acad Sci USA 2007 104 19193 8 10.1073/pnas.0707962104 18040045 PMC2148266

[B10] Oh VM The journal numbers game Ann Acad Med Singapore 2006 35 846 7 17218993

[B11] Cheek J Garnham B Quan J What's in a number? Issues in providing evidence of impact and quality of research(ers) Qualitative Health Res 2006 16 423 35 10.1177/1049732305285701 16449691

[B12] Hönekopp J Kleber J Sometimes the impact factor outshines the H index Retrovirology 2008 5 88 10.1186/1742-4690-5-88 18837971 PMC2569067

[B13] Web of Science http://thomsonreuters.com/products_services/science/science_products/a-z/web_of_science/

[B14] Scopus http://www.scopus.com/home.url

[B15] Google Scholar http://www.scholar.google.com

[B16] Lehmann S Jackson AD Lautrup BE Measures for measures Nature 2006 444 1003 4 10.1038/4441003a 17183295

[B17] Jeang KT Impact factor, H index, peer comparisons, and Retrovirology: is it time to individualize citation metrics? Retrovirology 2007 4 42 10.1186/1742-4690-4-42 17577403 PMC1894640

[B18] Jeang KT H-index, mentoring-index, highly-cited and highly-accessed: how to evaluate scientists? Retrovirology 2008 5 106 10.1186/1742-4690-5-106 19032780 PMC2607307

[B19] Lee J Kraus KL Couldwell WT Use of the h index in neurosurgery. Clinical article J Neurosurg 2009 111 387 92 10.3171/2008.10.JNS08978 19392590

[B20] Spearman CM Quigley MJ Quigley MR Wilberger JE Survey of the h index for all of academic neurosurgery: another power-law phenomenon? J Neurosurg 2010 113 929 33 10.3171/2010.4.JNS091842 20469986

[B21] Ponce FA Lozano AM Academic impact and rankings of American and Canadian neurosurgical departments as assessed using the h index J Neurosurg 2010 113 447 57 10.3171/2010.3.JNS1032 20380531

[B22] Kulasegarah J Fenton JE Comparison of the h index with standard bibliometric indicators to rank influential otolaryngologists in Europe and North America Eur Arch Otorhinolaryngol 2010 267 455 8 10.1007/s00405-009-1009-5 19551398

[B23] Rad AE Brinjikji W Cloft HJ Kallmes DF The H-index in academic radiology Acad Radiol 2010 17 817 21 10.1016/j.acra.2010.03.011 20471868

[B24] Healy NA Glynn RW Scutaru C Groneberg D Kerin MJ Sweeney KJ The h index and the identification of global benchmarks for breast cancer research output Breast Cancer Res Treat 2011 10.1007/s10549-011-1436-z 21399892

[B25] Hunt GE Cleary M Walter G Psychiatry and the Hirsch h-index: The relationship between journal impact factors and accrued citations Harv Rev Psychiatry 2010 18 207 19 10.3109/10673229.2010.493742 20597591

[B26] Benway BM Kalidas P Cabello JM Bhayani SB Does citation analysis reveal association between h-index and academic rank in urology? Urology 2009 74 30 3 10.1016/j.urology.2008.10.045 19567283

[B27] Kelly CD Jennions MD H-index: age and sex make it unreliable Nature 2007 449 403 17898745 10.1038/449403c

[B28] Kelly CD Jennions MD The h index and career assessment by numbers Trends Ecol Evol 2006 21 167 70 10.1016/j.tree.2006.01.005 16701079

[B29] Mishra DC Citations: rankings weigh against developing nations Nature 2008 451 244 18202623 10.1038/451244a

[B30] Rousseau R New developments related to the Hirsch index Science Focus 2006 1 23 25

[B31] Dodson MV Citation analysis: Maintenance of h-index and use of e-index Biochem Biophys Res Commun 2009 387 625 6 10.1016/j.bbrc.2009.07.091 19632203

[B32] Zhang CT The e-index, complementing the h-index for excess citations PLoS One 2009 4 e5429 10.1371/journal.pone.0005429 19415119 PMC2673580

[B33] Thomaz PG Assad RS Moreira LF Using the impact factor and H index to assess researchers and publications Arq Bras Cardiol 2011 96 90 93 10.1590/S0066-782X2011000200001 21445465

[B34] Allen L Jones C Dolby K Lynn D Walport M Looking for landmarks: the role of expert review and bibliometric analysis in evaluating scientific publication outputs PLoS One 2009 4 e5910 10.1371/journal.pone.0005910 19536339 PMC2695409

[B35] Bornmann L Daniel HD The state of h index research. Is the h index the ideal way to measure research performance? EMBO Rep 2009 10 2 6 10.1038/embor.2008.233 19079129 PMC2613214

[B36] Todd PA Ladle RJ Citations: poor practices by authors reduce their value Nature 2008 451 244 18202622 10.1038/451244b

[B37] Ball P Achievement index climbs the ranks Nature 2007 448 737 10.1038/448737a 17700666

[B38] Balandin S Stancliffe RJ Impact factors and the h-index: what researchers and readers need to know Augment Altern Commun 2009 25 1 3 10.1080/07434610902813549 19280418

[B39] Williamson JR My h-index turns 40: my midlife crisis of impact ACS Chem Biol 2009 4 311 3 10.1021/cb9001014 19441857

[B40] Purvis A The h index: playing the numbers game Trends Ecol Evol 2006 21 422 10.1016/j.tree.2006.05.014 16781007

[B41] Kotov NA Fraud, the h-index, and Pasternak ACS Nano 2010 4 585 6 10.1021/nn100182y 20175562

